# Non-suicidal self-injury: state of the art perspective of a proposed new syndrome for DSM V

**DOI:** 10.1186/1753-2000-6-9

**Published:** 2012-03-30

**Authors:** Paul L Plener, Joerg M Fegert

**Affiliations:** 1 Department of Child and Adolescent Psychiatry and Psychotherapy, University of Ulm, Steinhoevelstr. 5, 89075 Ulm, Germany

## 

Non-suicidal Self-Injury (NSSI) has received an increasing amount of attention over the last years. Although first papers on NSSI date back to the 1960s, it was not until 2002, that the first epidemiological study on adolescents was published, leading to a large body of research that has been growing ever since. Ten years after this seminal study, that showed a high prevalence of NSSI in a non-clinical population [1], we are discussing a new entity, which has been proposed to be included in the DSM V [2] as Non-Suicidal Self-Injury Syndrome. This proposal was the result of seeing a growing number of adolescents, that did not fulfil the criteria for Borderline Personality disorder (the only diagnostic category in DSM IV and ICD-10 that listed NSSI as symptom), but were nevertheless distressed, in need of help and - as recent research showed - at risk for suicide. It has been discussed, whether a new diagnostic entity makes sense, especially with regards to suicide and the relationship between NSSI and suicidality [3-5]. However, given the high prevalence of adolescents that "use" NSSI as emotional regulation "skill" and clearly distance themselves from suicidality, the inclusion in a classificatory systems seems to make sense in order to foster further treatment and research in an area, that causes a lot of distress in adolescents, research that within the next years will have to focus on trajectories and predictors, since the first promising longitudinal studies, reported specific risk factors [6] and a strong decrease of prevalence in young adulthood [7], both of which needs to be understood more clearly in order to assist therapeutic approaches to treating NSSI.

As discussing the inclusion of NSSI as a syndrome in the DSM V, we have to keep in mind, that the fast increase in knowledge is the product of an international effort to more deeply understand a phenomenon that has been not only troubling adolescents, but also their families, teachers and clinicians as well. At the verge of the creation of a new diagnostic entity, our aim was to give a broad "state of the art" overview about NSSI. Since a diagnostic entity needs to be seen from different angles (namely: nomenclature, epidemiology, therapy, impact on society, ...), we tried to shed a light on NSSI, coming from different perspectives. As the International Society for the Study of Self-Injury (ISSS) has dedicated itself to research in this field, we asked several members to provide us with insights in their respective field of research. To start with, Muehlenkamp et al., present a systematic review about the prevalence of NSSI in adolescent samples. Within the last 7 years, 53 studies have been published, providing us with prevalence data. As the debate about the nomenclature of NSSI especially with regards to suicidal intent is ongoing, it is surprising, that both studies, that have used an NSSI nomenclature, as well as studies, that used a definition of Deliberate Self Harm (without explicitly excluding conscious suicidal intent), nearly showed the same prevalence rates. Given the mean prevalence rate of 18% reported for non-clinical adolescent populations in this paper, it seems clear, that NSSI has left it's imprint in adolescent culture and society. Andover et al., provide a review about the relationship between NSSI and attempted suicide. Despite their distinctiveness these behaviors are intertwined sharing both similar and separate associated factors. Research over the last years has suggested that NSSI has to be acknowledged as a risk factor for suicide attempts, thus underlining the clinical importance of NSSI assessment. It is a further sign of the currentness of NSSI, that while we were preparing this special issue, we received an original paper (Ferrara et al.) as well, providing new data about personality features in NSSI from a sample of 52 adolescent inpatients. Authors also reported on a reduced "attraction to life" disposition in adolescents with NSSI, thus closing the circle to the review from Andover et al. Lewis & Heath reflect on NSSI and the new media. Dissemination via the World Wide Web has been discussed as one of the possible reason of the rapid spread of NSSI and the authors' former work has resulted in You Tube reflecting their policy on NSSI video content, thus showing the impact research in this area can have. They provide the reader with both knowledge and strategies for assessment. Given that NSSI experiences that are shared on the web, present a risk for NSSI reinforcement, the authors urge the clinician to thoroughly assess their patients' online behavior and provide recommendations for doing so as well as listing several helpful sites to assist in tackling NSSI. Finally, the paper by Washburn and colleagues deals with therapeutic approaches to treating NSSI, thus showing, that at this point, we already know ways how to treat NSSI, thus adding further support to a diagnostic entity NSSI, as it represents not only a nomenclatory issue, but a treatable condition. However, most of evidence based research in this field stems from studies on adults, thus showing the need for treatment approaches directed towards adolescents.

This special issue is a truly international effort made possible by a grant of the German research foundation, with authors from the USA, Canada, Belgium, Italy and Germany, showing that although work on NSSI has been largely done in the US within the last years, the field is growing tremendously towards a global research effort. Apart from presenting our respectable readership state of the art reviews, we are now able to deliver a special issue that combines both, reviews, representing past research and an original paper adding further knowledge to support a better understanding of NSSI, thus showing research on NSSI as it is: a work in progress.
